# Society Meetings

**Published:** 1908-12

**Authors:** 


### SOCIETY MEETINGS.

---

The New York and New England Association of Railway Surgeons held its eighteenth annual meeting at New York, November 17, 1908, under the presidency of Dr. F. A. Stillings. In his annual address, Dr. Stillings advised medical men connected with railways not to ride on passes given in payment for their services. He said surgeons should avoid contract service with railways unless they received enough salary to devote all their time to the work.

Officers were elected as follows: president, Dr. J. M. Wainwright, Scranton, Pa.; vice-presidents, Dr. C. A. Pease, Burlington, Vt., and Dr. G. C. Madill, Ogdensburg; corresponding secretary, Dr. George Chaffee, Brooklyn; recording secretary, Dr. C. B. Herrick, Troy; treasurer, Dr. J. K. Stockwell, Oswego.

---

The Medical Association of Central New York met Tuesday, October 27, 1908, in the Buffalo Historical Society building, under the presidency of Dr. Carlton C. Frederick, of Buffalo. The president's address, dealing with the topic of anesthesia is published elsewhere in this issue. Other papers will appear in due course. Officers were elected as follows: president, R. H. Conway, Auburn; first vice-president, F. H. Stevenson, Syracuse; second vice-president, E. W. Mulligan, Rochester; secretary, C. A. Greenleaf, Hornell (reëlected); treasurer, C. O. Boswell, Rochester. The next meeting will be held in October, 1909, at Auburn.

---

THE Buffalo Academy of Medicine held meetings during November as follows:

*Section of Surgery.*—The regular meeting of this section was held Monday evening, November 2, 1908 at 8.30. Program: (1) "The diagnosis of extrauterine pregnancy. and the report of a case of primary ovarian pregnancy." James A. McLeod; (2) Demonstration on throat surgery, Geo. F. Cott; (3) Microscopical demonstration of opsonic indices, Norman K. McLeod; (4) Muroscopal demonstration of decidual cells in extrauterine pregnancy, Charles A. Bentz.

*Section of Medicine.*—The regular meeting of this section was held Tuesday evening, November 10, 1908, at 8.30. Program: (1) The post-operative prognosis of spinal cord tumors, William C. Krauss.

*Section of Pathology.*—The regular meeting of this section was held Tuesday evening, November 17, 1908, at 8.30. Program: (1) Darier's disease ending in multiple skin cancer, Grover W. Wende, Dr. Wende exhibited specimens and presented the patients. (2) Cancer and sarcoma. A series of experiments made for the purpose of determining the origin and cause of cancer and sarcoma, their methods of propagation, means of prevention and also a few remarks upon their cure, H. D. Walker, Newburg, N. Y.

*Section of Obstetrics and Gynecology.*—A special stated meeting of this section was held Tuesday evening, November 24, 1908, at 8.30. Program: The choice of operation for uterine and bladder displacement and prolapse, Isaac S. Stone, Washington, D. C. Discussion opened by C. C. Frederick.

The Buffalo Academy of Medicine was incorporated November 10, 1908. This meeting was called to adopt Constitution and

By-Laws, and to hear a supplementary report of the Contagious Hospital Commission.

---

THE Rochester Academy of Medicine held regular monthly meetings at Hotel Seneca as follows:

Wednesday evening, November 11.—Candidate for resident fellowship, Irving L. Walker. Program: Some recent aspects of the cancer problem, Roswell Park, of Buffalo.

Wednesday evening, November 18. Program: The dead man's curve, Thomas Jameson.

Wednesday evening, November 25. Program: The prevention of ophthalmia neonatorum. Illustrated by lantern. F. Park Lewis of Buffalo.

Friday evening, December 4. Program: The Comparative values of the tuberculin tests, with special consideration of the ophthalmic, Henry L. Elsner, Syracuse.

